# Supplementary material for: Protective effects of ectoine on articular chondrocytes and cartilage in rats for treating osteoarthritis
Source: PLoS One. 2024 Feb 29;19(2):e0299351. doi: 10.1371/journal.pone.0299351 (PMC10903896; doi:10.1371/journal.pone.0299351)
Supplement: S1 File — (PDF) [file pone.0299351.s001.pdf]

Chondrocytes treated with 0% ectoine and tyrpsin

Test 1 Test 2

0 min

2 min

Test 3 Test 4

0 min

2 min

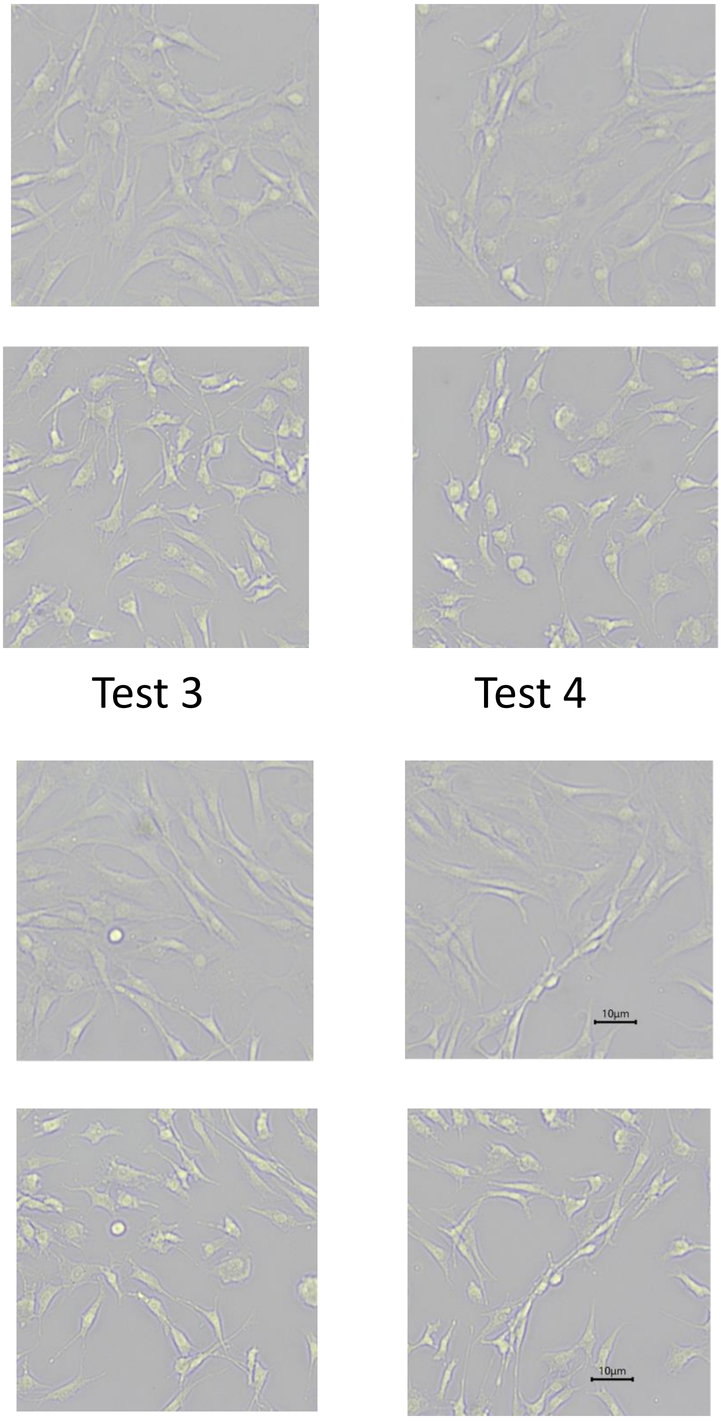

Scare bar= 10um

Chondrocytes treated with 0.5% ectoine and tyrpsin

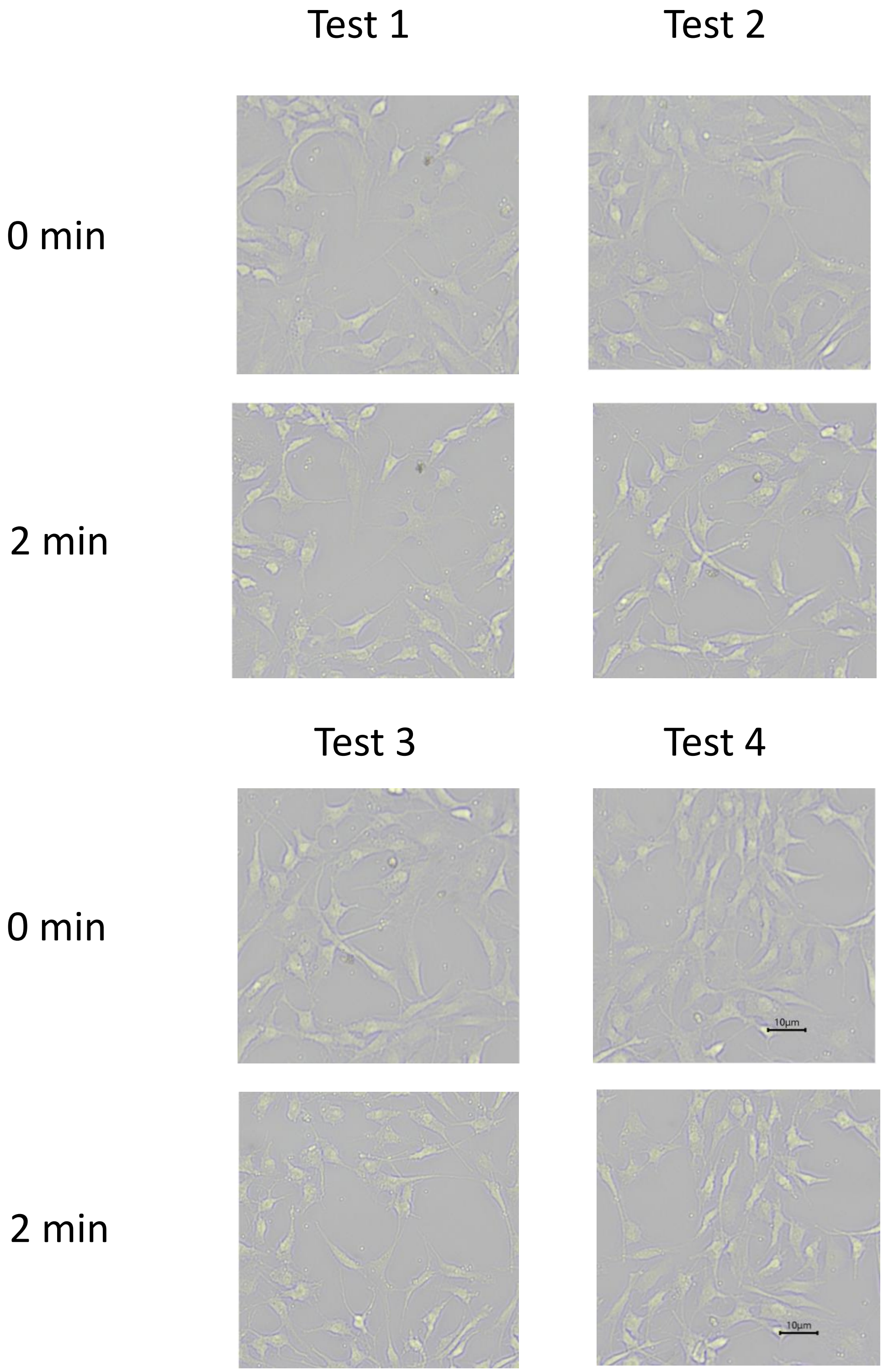

Scare bar= 10um

Chondrocytes treated with 1.0% ectoine and tyrpsin

Test 1 | Test 2

0 min

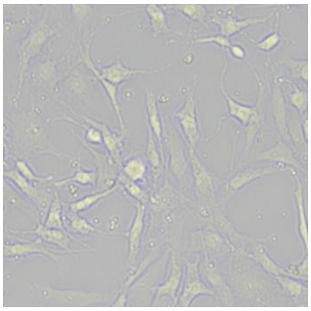

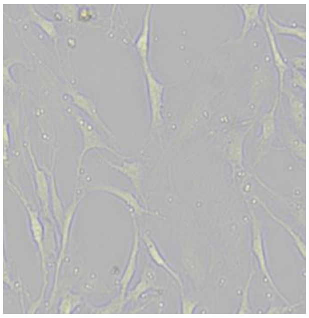

2 min

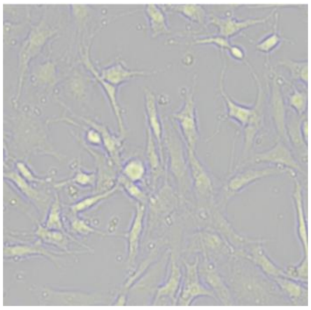

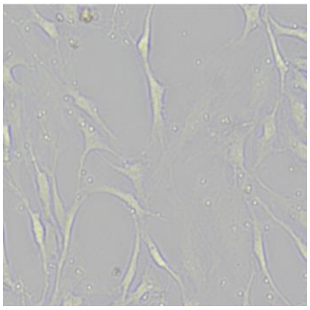

Test 3 | Test 4

0 min

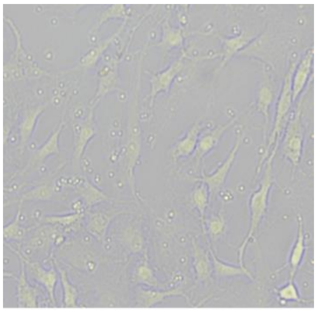

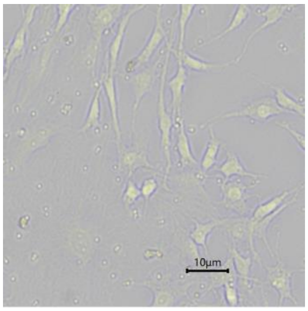

2 min

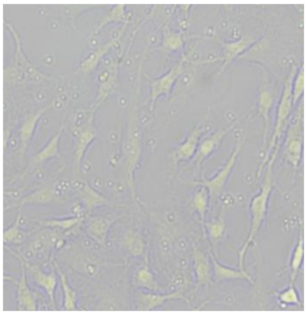

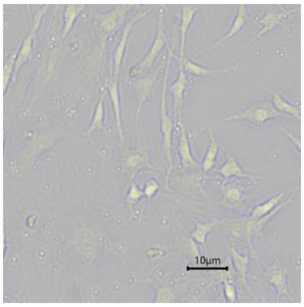

Scare bar= 10um

Chondrocytes treated with 1.5% ectoine and tyrpsin

Test 1 Test 2

0 min

2 min

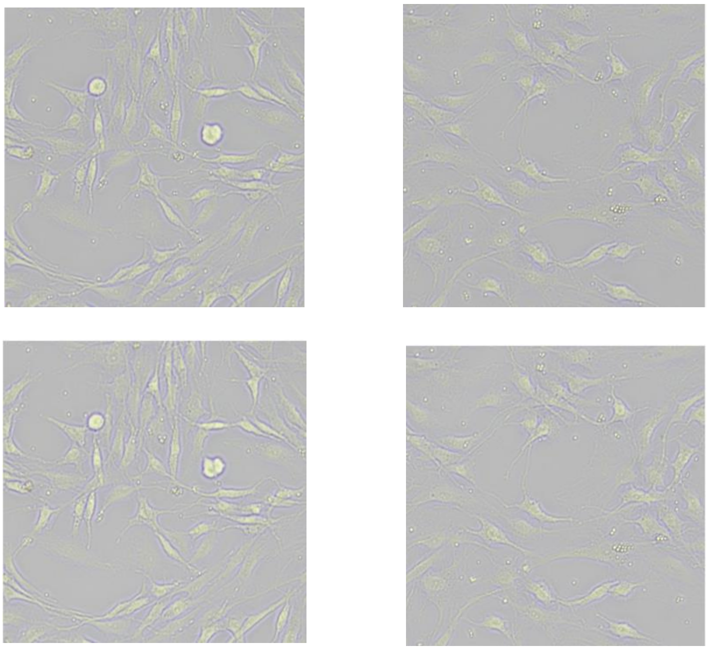

Test 3 Test 4

0 min

2 min

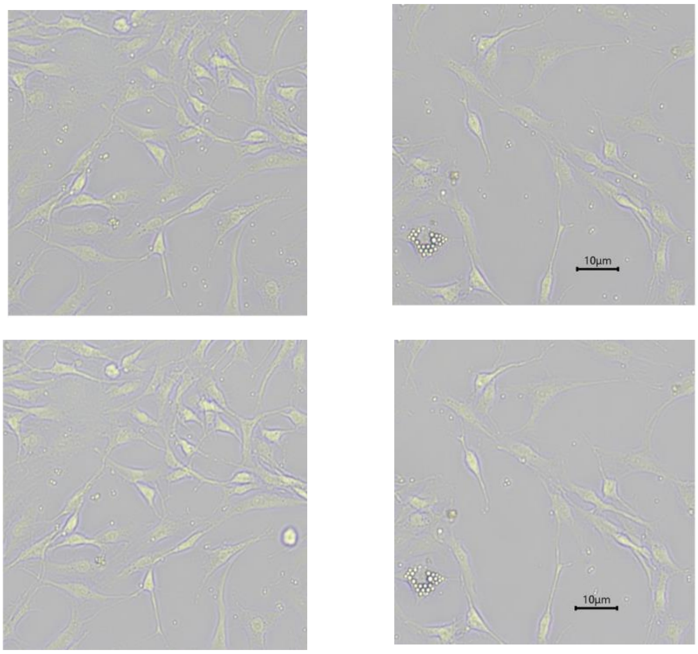

Scare bar= 10um
