# Supplementary material for: Protective effects of ectoine on articular chondrocytes and cartilage in rats for treating osteoarthritis
Source: PLoS One. 2024 Feb 29;19(2):e0299351. doi: 10.1371/journal.pone.0299351 (PMC10903896; doi:10.1371/journal.pone.0299351)
Supplement: S2 File — (PDF) [file pone.0299351.s002.pdf]

ROS images

Control | H2O2

Test 1

Test 2

Test 3

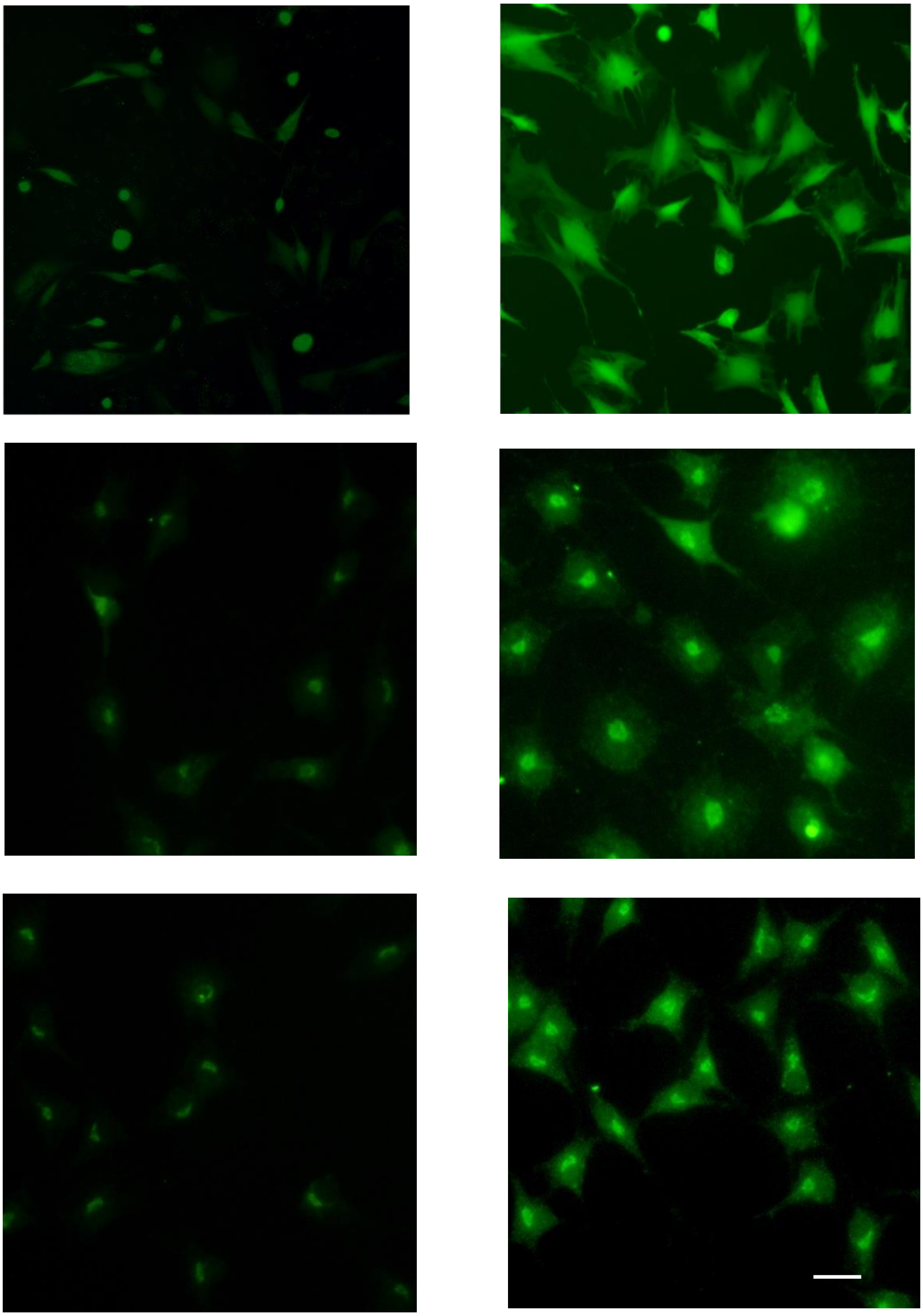

Scare bar= 10um

ROS images

0.5%Ec+H2O2 | 1.0%Ec+H2O2

Test 1

Test 2

Test 3

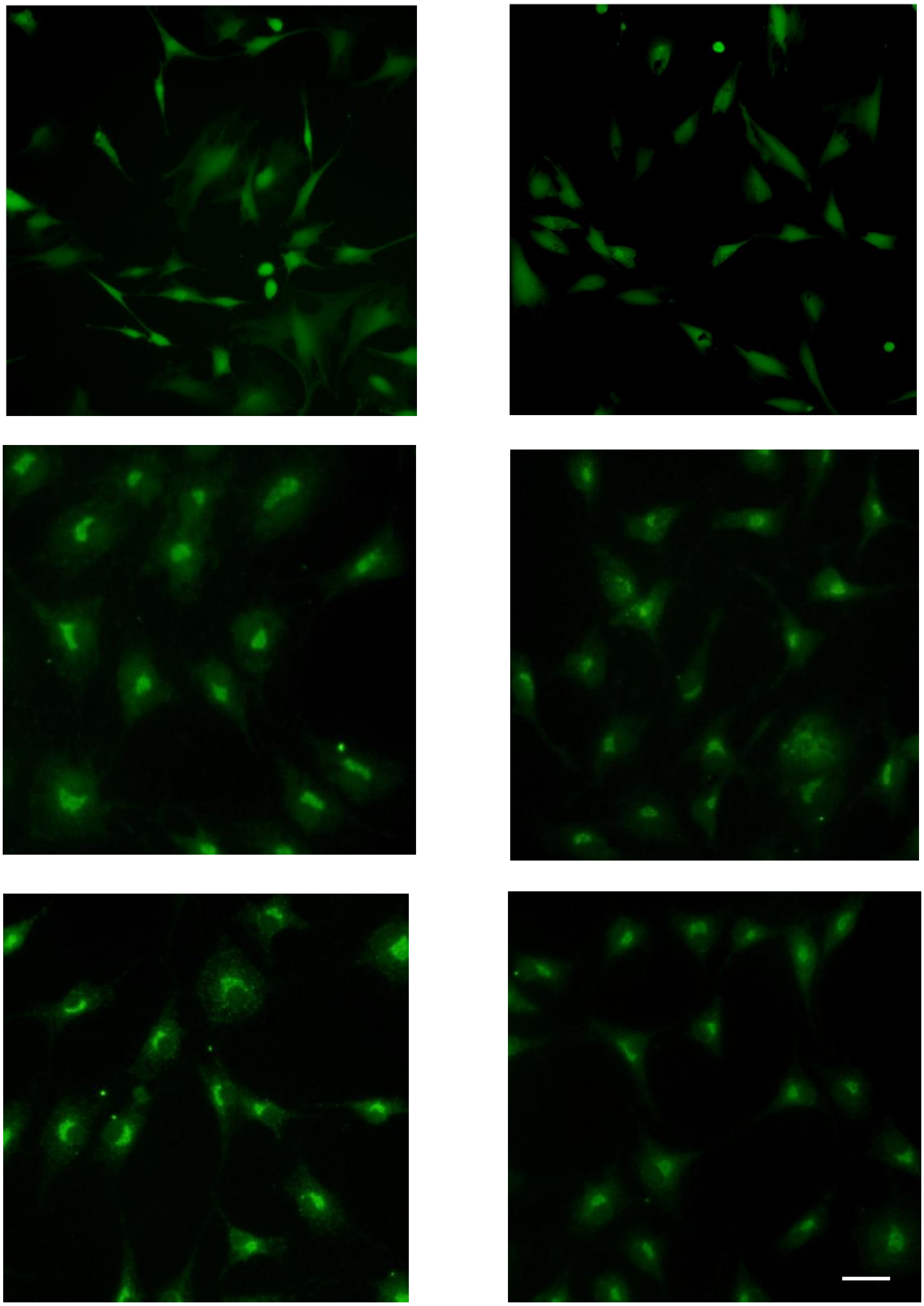

Scare bar= 10um

ROS images

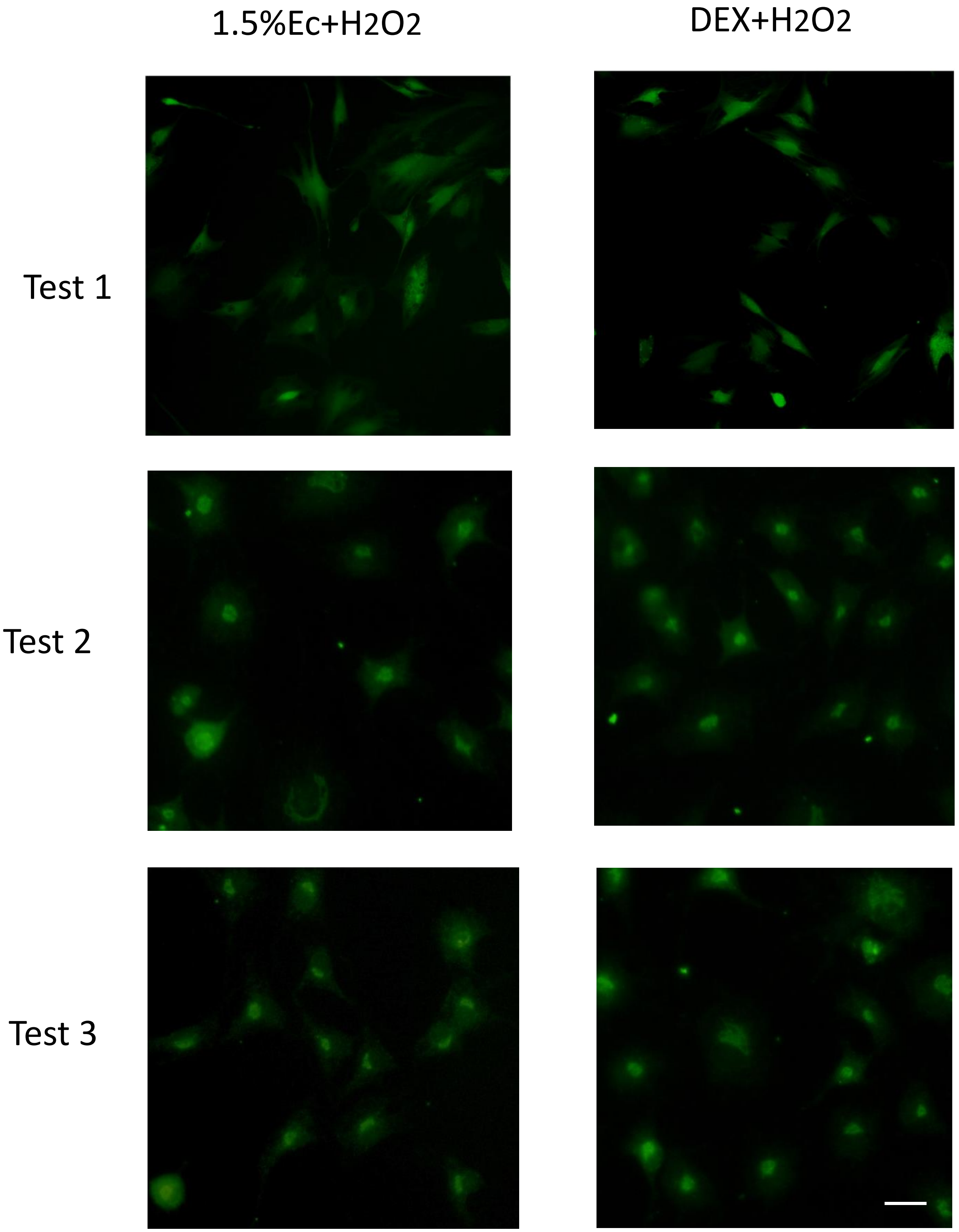

Scare bar= 10um
