# Supplementary material for: Protective effects of ectoine on articular chondrocytes and cartilage in rats for treating osteoarthritis
Source: PLoS One. 2024 Feb 29;19(2):e0299351. doi: 10.1371/journal.pone.0299351 (PMC10903896; doi:10.1371/journal.pone.0299351)
Supplement: S3 File — (PDF) [file pone.0299351.s003.pdf]

Immunofluorescence staining of type II collagen

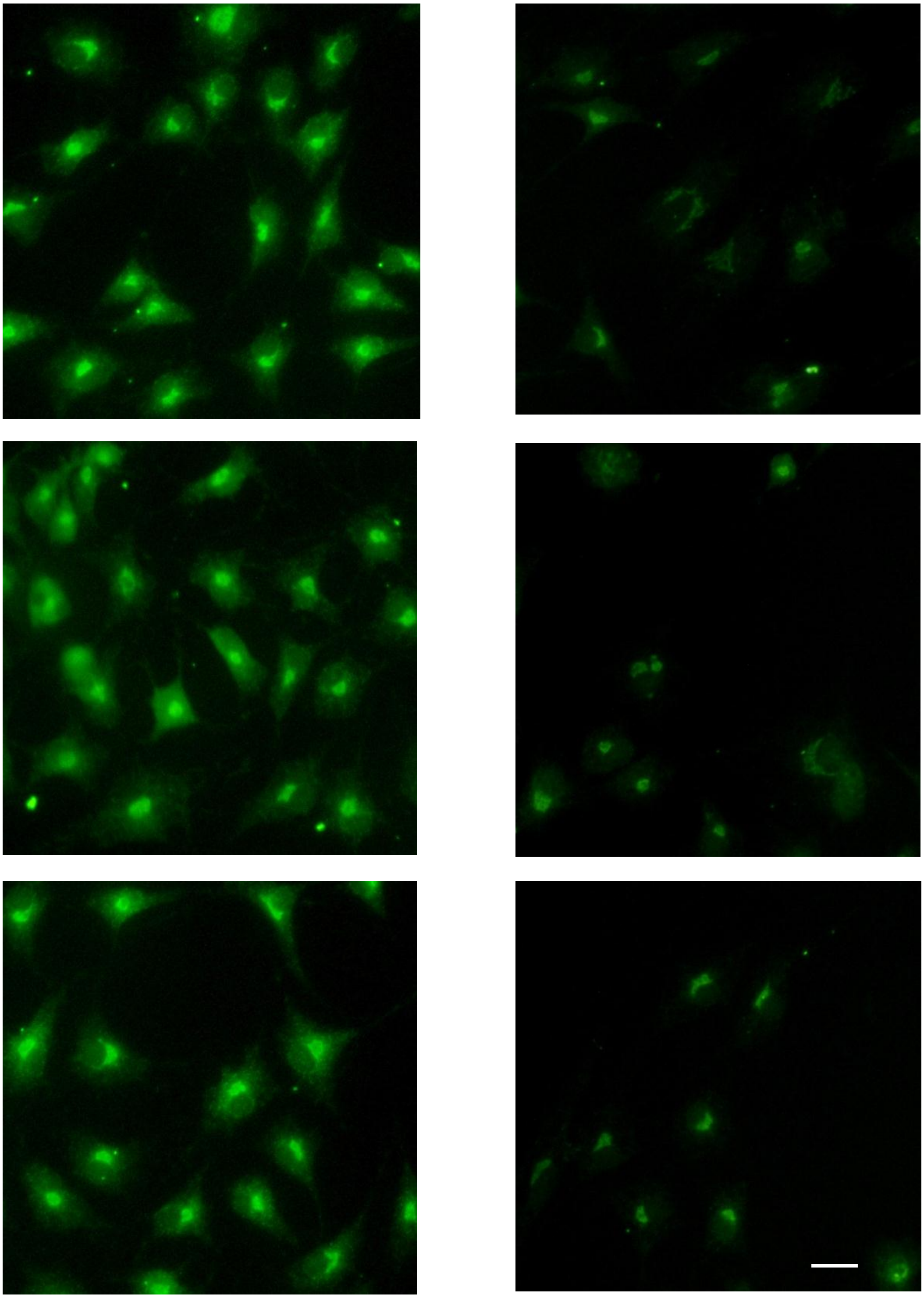

Scare bar= 10 um

Immunofluorescence staining of type II collagen

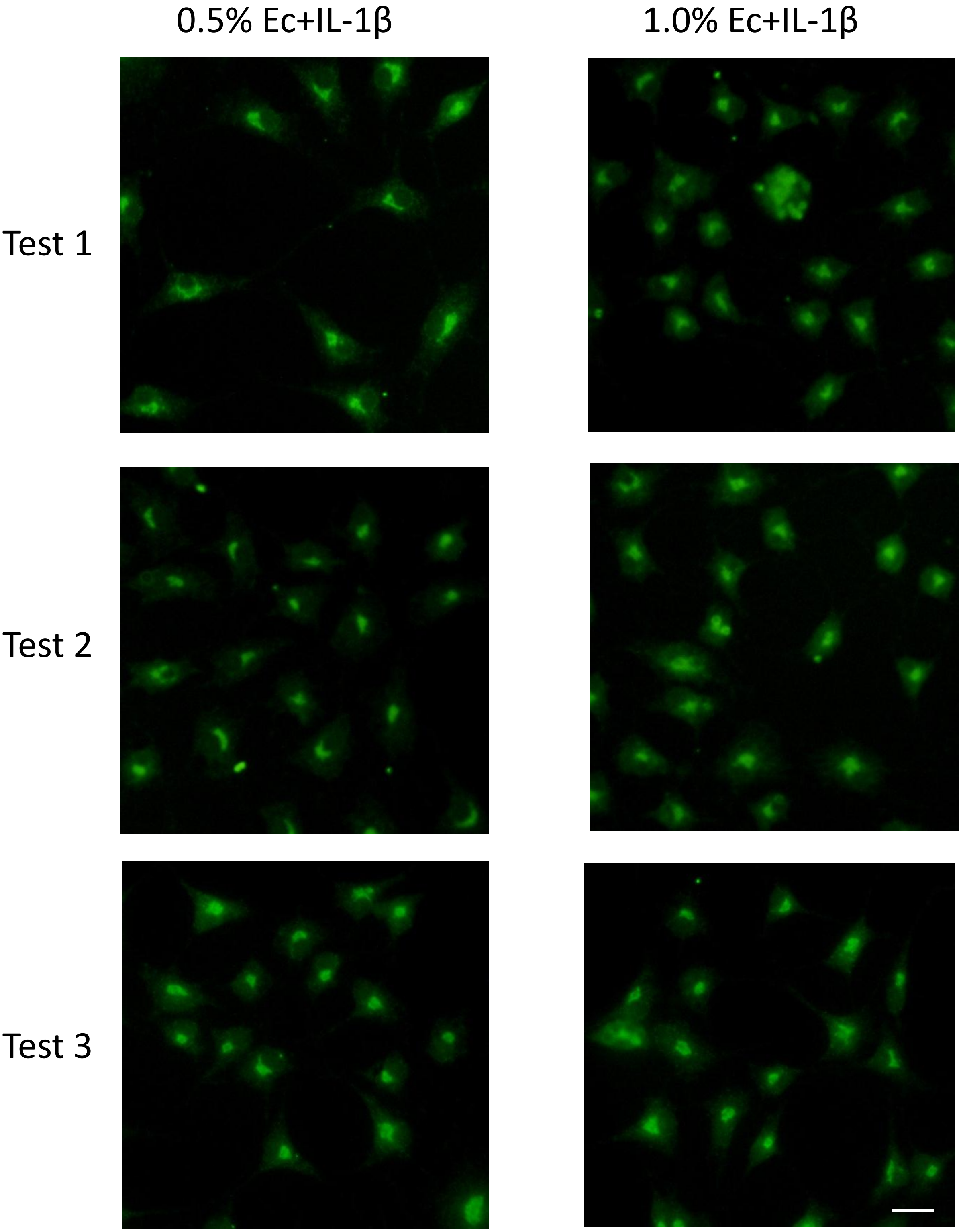

Scare bar= 10 um

Immunofluorescence staining of type II collagen

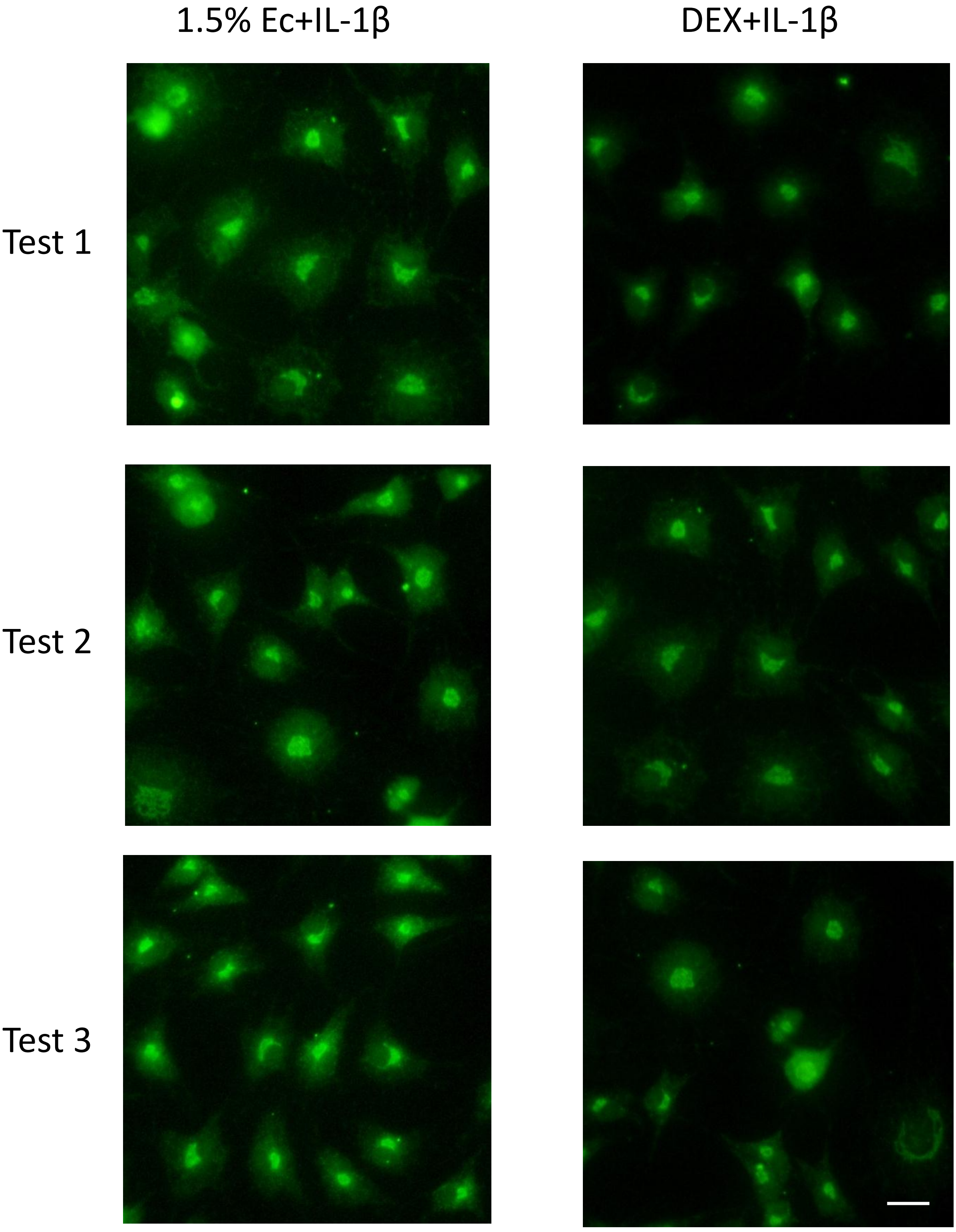

Scare bar= 10 um
