# Supplementary material for: Protective effects of ectoine on articular chondrocytes and cartilage in rats for treating osteoarthritis
Source: PLoS One. 2024 Feb 29;19(2):e0299351. doi: 10.1371/journal.pone.0299351 (PMC10903896; doi:10.1371/journal.pone.0299351)
Supplement: S4 File — (PDF) [file pone.0299351.s004.pdf]

H-E and S-O staining of cartilage

Sham group

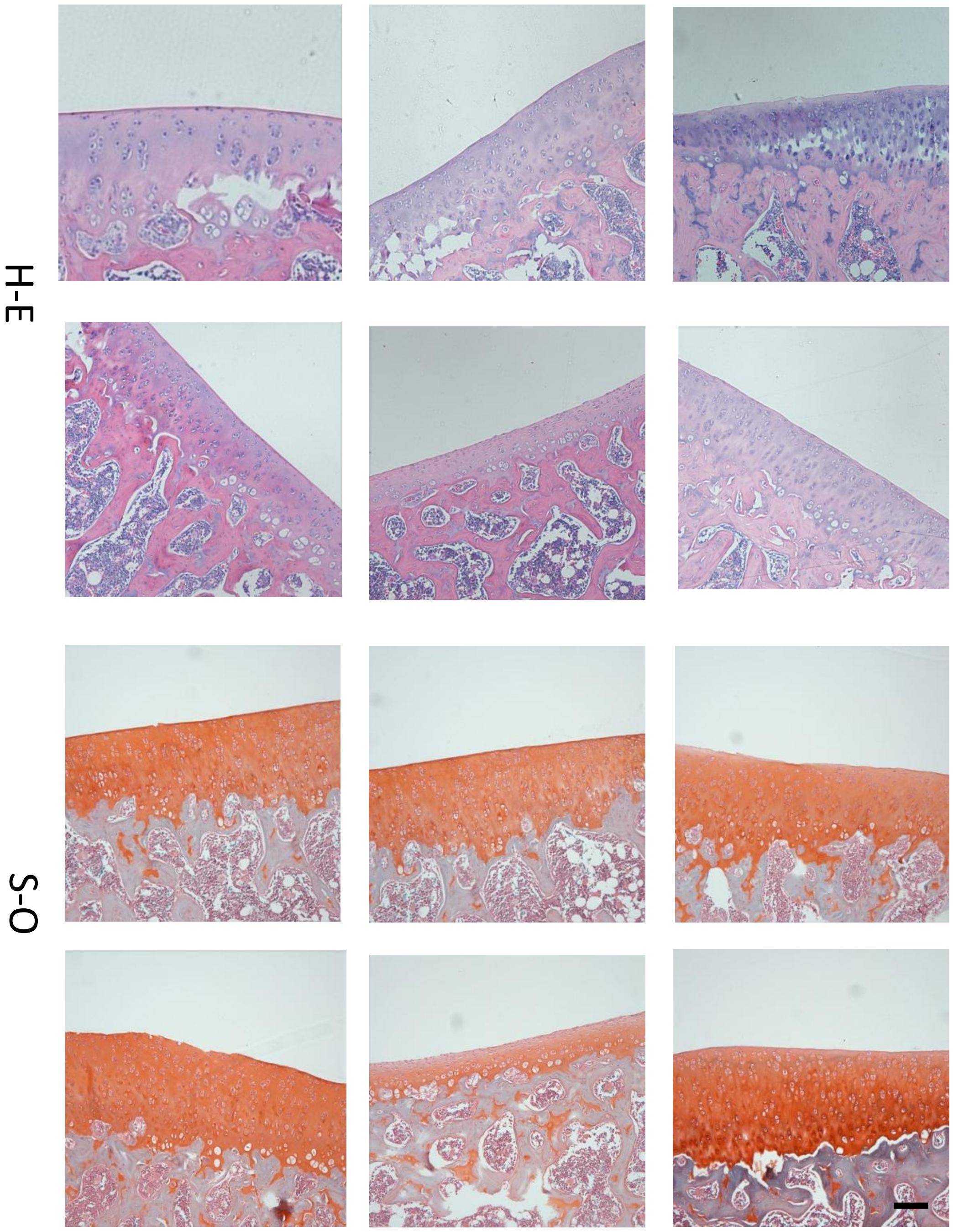

Scare bar= 100um

H-E and S-O staining of cartilage

OA group

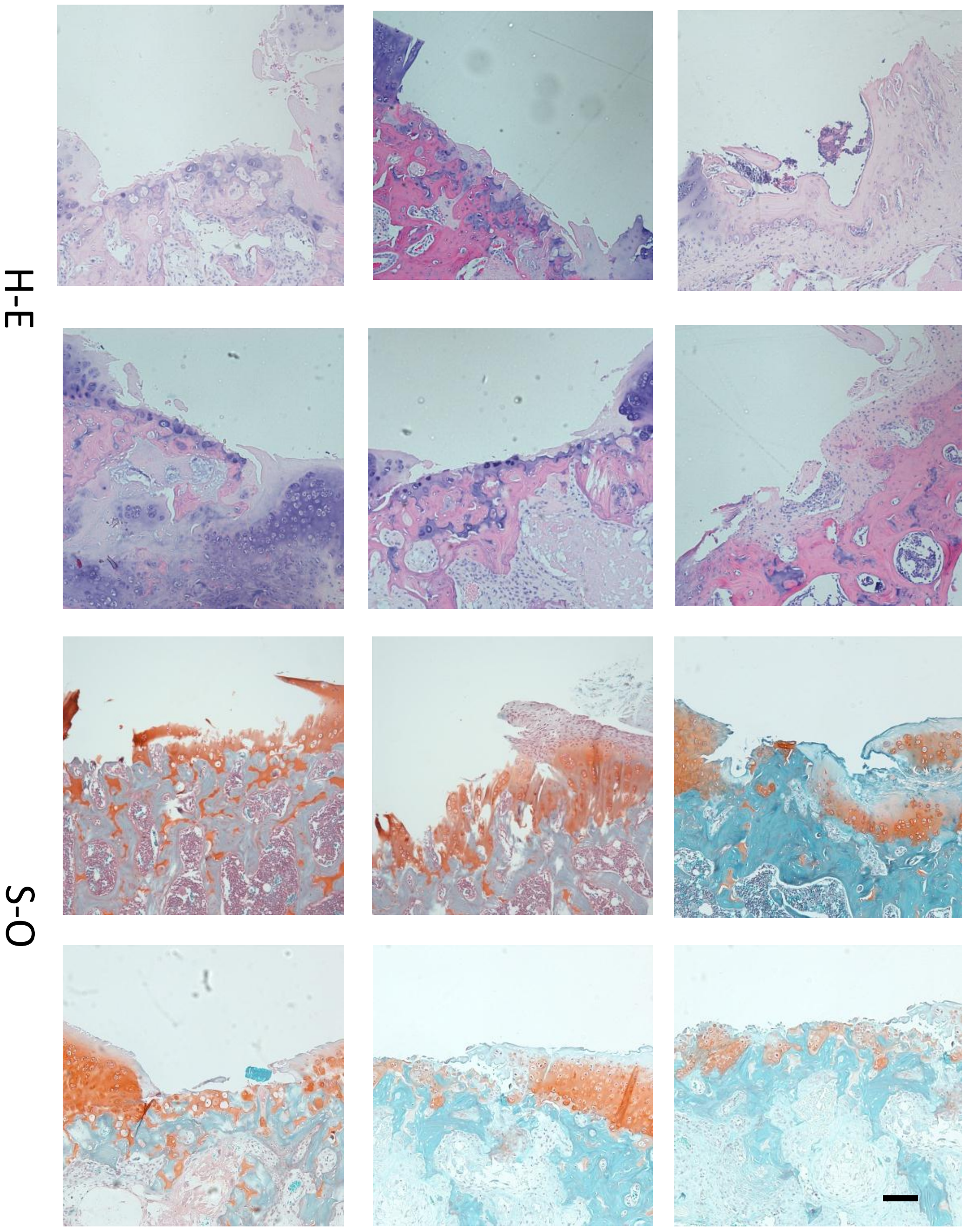

Scare bar= 100um

H-E and S-O staining of cartilage

OA+Ec group

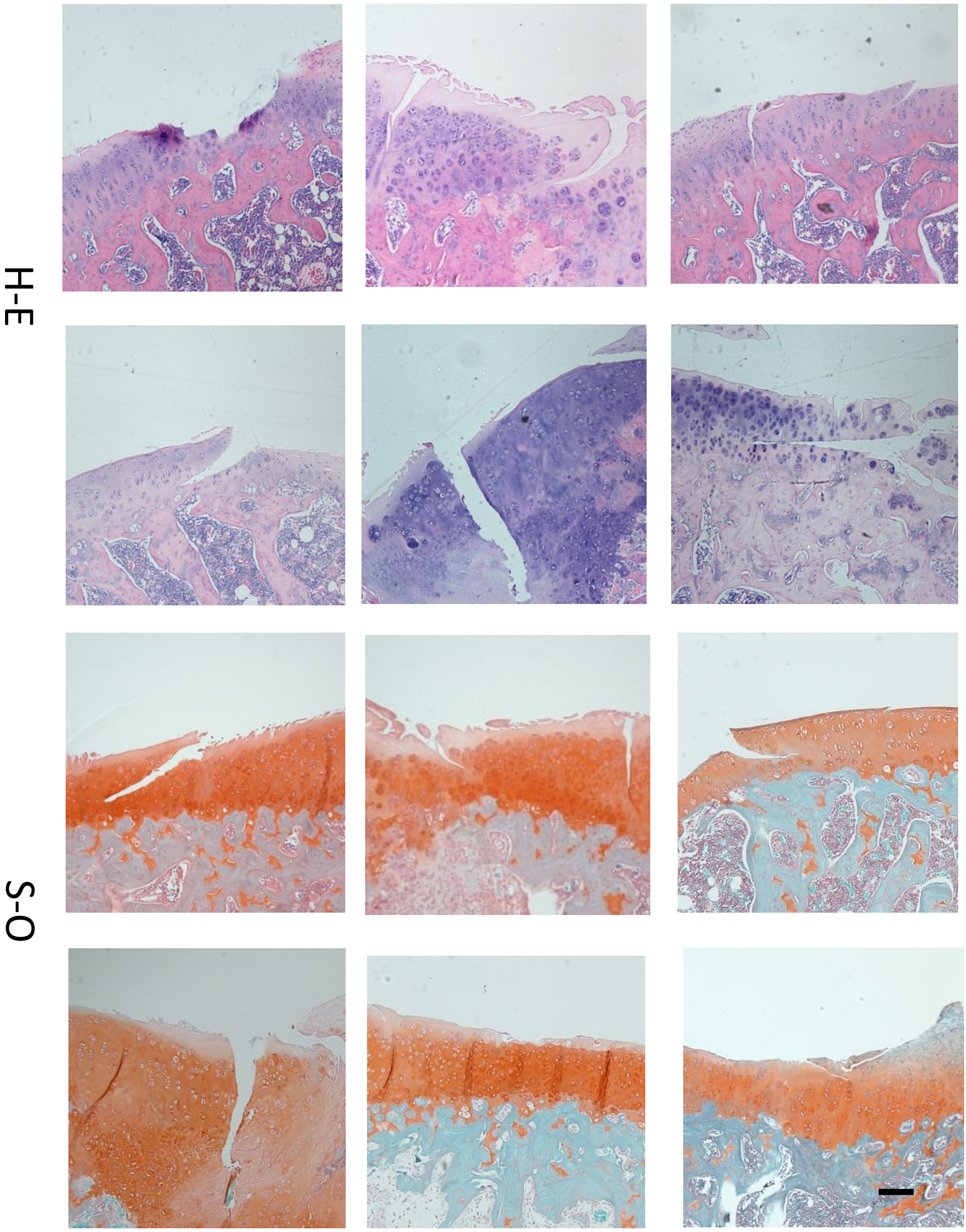

Scare bar= 100um

H-E and S-O staining of cartilage

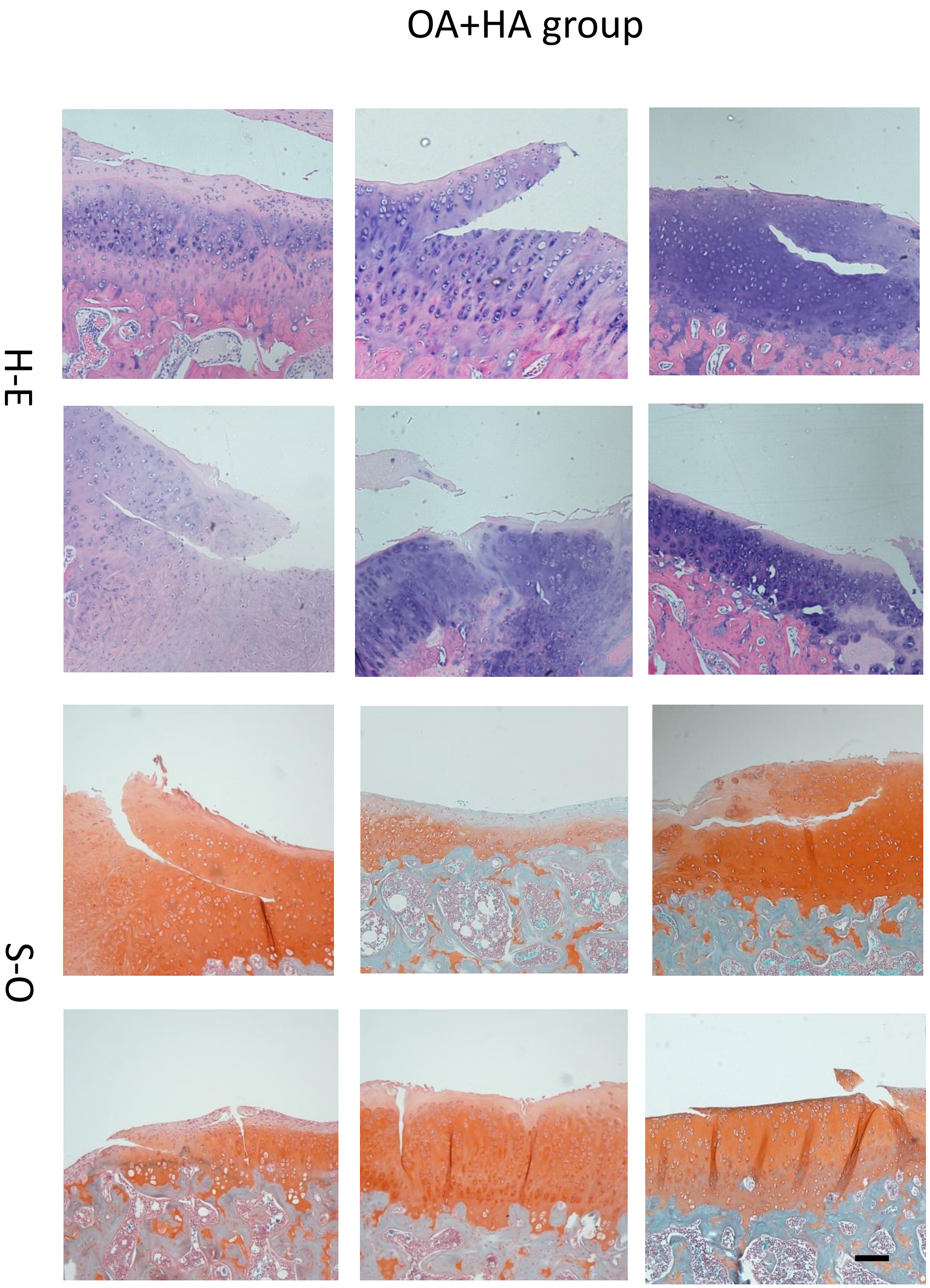

Scare bar= 100um
