# Supplementary material for: Protective effects of ectoine on articular chondrocytes and cartilage in rats for treating osteoarthritis
Source: PLoS One. 2024 Feb 29;19(2):e0299351. doi: 10.1371/journal.pone.0299351 (PMC10903896; doi:10.1371/journal.pone.0299351)
Supplement: Attachment — Submitted filename: plos one.docx [file pone.0299351.s011.docx]

Addressing osteoarthritis (OA), characterized by articular cartilage degradation and inflammation. In vitro experiments assessed the impact of ectoine on chondrocytes' trypsin resistance, viability at elevated temperatures, and resistance to oxidative stress. The study also examined ectoine's effects on chondrocytes treated with IL-1β, measuring gene expression levels of key markers and assessing type II collagen synthesis. In an in vivo rat OA model, ectoine demonstrated protective effects, reducing cartilage degeneration and Osteoarthritis Research Society International (OARSI) scores. Overall, the findings suggest that ectoine may serve as a potential therapeutic agent for OA by safeguarding chondrocytes and preserving cartilage integrity. I recommend the manuscript for the publication but it require to answer certain questions.

Why does articular cartilage have a weak self-repairing ability after injury, and what are the limitations of current treatments such as debridement, lavage, microfracture, and autologous chondrocyte implantation?

In what ways does the study suggest that ectoine could be beneficial in the field of cartilage repair and OA treatment? Include this in the introduction part.

Experimental Design and Methods:

a. How were the chondrocytes initially prepared and treated with trypsin in the in vitro study?

b. Can you provide more details on the MTT assay used to assess the effect of ectoine on the heat resistance of chondrocytes?

c. What specific ROS species were measured in the ROS assay, and how were they quantified?

d. Were there any control groups in the in vitro experiments to validate the specificity of the observed effects of ectoine?

e. Could you elaborate on the rationale for choosing 50 °C as the temperature for the high-temperature resistance experiment?

Results and Data Interpretation:

a. In Figure 2, what might explain the difference in chondrocyte morphology between the ectoine groups after trypsin treatment?

b. Can you discuss the potential reasons for the observed dose-dependent effect of ectoine on trypsin resistance in chondrocytes?

c. How do you interpret the variations in cell viability at 50 °C with different concentrations of ectoine in Figure 3?

d. Regarding the ROS assay (Figure 4), what implications do the differences in fluorescence intensity between ectoine groups and the DEX group have for the study's objectives?

e. What are the possible mechanisms behind the observed inhibitory effects of ectoine on COX-2, MMP-3, and MMP-9 expression in chondrocytes (Figure 5)?

Future Directions and Implications:

a. In the discussion, you mention potential applications of ectoine in cartilage injury repair and tissue engineering. Can you elaborate on specific strategies or technologies where ectoine could be integrated?

b. What further studies or experiments would you recommend to elucidate the specific mechanisms through which ectoine exerts its protective effects on chondrocytes?

c. How might the findings of this study inform the development of novel therapeutic interventions for OA, and what are the potential challenges in translating these findings to clinical practice?
